# The Extent and Nature of Autistic People’s Violence Experiences During Adulthood: A Cross-sectional Study of Victimisation

**DOI:** 10.1007/s10803-022-05647-3

**Published:** 2022-07-11

**Authors:** Vicki Gibbs, Jennie Hudson, Elizabeth Pellicano

**Affiliations:** 1 Macquarie School of Education, Macquarie University, Sydney, Australia; 2 Black Dog Institute, Sydney, Australia

**Keywords:** Autism, Autistic adults, Violence, Victimisation

## Abstract

This study investigated the extent and nature of violence experiences reported by autistic adults. Autistic (n = 118) and non-autistic (n = 110) adults completed a questionnaire about their experiences of sexual harassment, stalking and harassment, sexual violence and physical violence since the age of 15. Autistic adults reported higher rates of all violence types, multiple forms of violence and repeated instances of the same type of violence. Typical gender differences in the patterns of violence (more physical violence reported by men and more sexual violence reported by women) were apparent in the non-autistic but not the autistic group. Findings add to the limited research in this area and highlight the need to identify risk and protective factors. Policy and practice implications are also discussed.

**Supplementary Information:**

The online version contains supplementary material available at 10.1007/s10803-022-05647-3.

## Introduction

Disabled people are at significantly higher risk of experiencing sexual and physical violence during their lifetime than people without disabilities (Codina et al., 2022; Krnjacki et al., 2016; Mailhot Amborski et al., 2021). The degree of elevated risk varies across disability type, with those who have an intellectual disability or mental illness experiencing the highest rates of violence and abuse (Byrne, 2018; Fisher et al., 2016; Nixon et al., 2017). There is now a substantial research base investigating risk factors, sequelae and prevention strategies for these vulnerable populations (e.g., Byrne, 2018; de Vries et al., 2019; McGilloway et al., 2020; Morgan et al., 2016). Fewer studies have examined violence exposure for autistic people, particularly those without co-occurring intellectual disability.

To date, most research investigating violence amongst autistic people has been based on parent report and largely focused on peer victimisation and abuse during childhood—consistently showing that autistic children experience higher rates of bullying, assault and child maltreatment compared to typical children (Hellström, 2019; Mandell et al., 2005; McDonnell et al., 2019; Pfeffer, 2016). There is emerging evidence that this increased risk continues well into adulthood and extends to criminal victimisation such as physical and sexual assault. Studies that have gathered first-hand information about sexuality, adverse experiences and trauma over the lifespan from autistic adults have found high-rates of sexual and physical violence (Brown-Lavoie et al., 2014; Hartmann et al., 2019; Pecora et al., 2019, 2020; Reuben et al., 2021; Rumball et al., 2020), although it is unclear from these studies whether the violence occurred during childhood, adulthood or both.

Only three studies have included measures with questions specifically pertaining to violence experienced during adulthood. In one large international study, autistic adults reported significantly higher rates of coerced and forced sexual activity, were more likely to have been sexually abused or threatened by a relationship partner and were more likely to be physically assaulted during their adult years compared to non-autistic adults (Griffiths et al., 2019). Similarly, Weiss and Fardella (2018) found that rates of sexual assault and physical assault with a weapon during adulthood were higher for autistic compared to non-autistic adults. In contrast, Gibbs et al. (2021) found no difference in reported physical and sexual violence during adulthood for autistic and non-autistic adults, although this information pertained to the previous two years only.

Bronfenbrenner’s ecological model (1977) has been proposed as a model for understanding factors that may contribute towards interpersonal violence amongst autistic people (Fardella et al., 2018). This model proposes that victimisation is the result of a complex interplay of four levels of factors i.e. ontogenic development (individual factors or experiences), the microsystem (the immediate context in which the victimization occurs, intimate and family relationships etc.), the exosystem (such as school, workplace, community supports) and the macrosystem (broader societal structures/cultural norms). At the ontogenic and microsystem levels, individual characteristics associated with autism such as social-communication differences may make it difficult to recognise dangerous situations or manipulation and coercion by others and lead to increased isolation and fewer protective relationships, all of which may contribute to disproportionately high rates of violence for autistic people (Kerns et al., 2015; Paul et al., 2018; Roberts et al., 2015). At the exosystem and macrosystem levels, socio-cultural factors such as stigma and discrimination often associated with autism (Botha & Frost, 2020; Shtayermman, 2009), inaccurate perceptions of autistic people as inferior or incapable (Heasman & Gillespie, 2018) and negative evaluations of autistic people by others (Sasson et al., 2017) may further increase the likelihood of an autistic person being targeted by perpetrators, who often choose victims perceived as weaker, less able to defend themselves and/or less likely to make accusations against them (Nettleback & Wilson, 2002).

In the neurotypical population, men report more physical violence and women more sexual assaults, intimate partner violence and stalking (Lauritsen & Carbone-Lopez, 2011; US Department of Justice, 2019). These gender differences have been found to be narrowed amongst people with intellectual disability (Platt et al., 2017) and mental illness (de Mooji et al., 2015). Transgender people and those with non-conforming gender identities (TGNC) have been found to have elevated rates of sexual victimisation, harassment and stalking compared to cisgender people (Fedina et al., 2020; Newcomb et al., 2020). Most studies investigating violence in autistic adults have focused on sexual victimisation of women or have consisted of small sample sizes which have not allowed for gender comparisons. One study found higher rates of both sexual and physical violence for autistic women compared to autistic men (Gibbs et al., 2021). Pecora et al. (2020) found no evidence of increased unwanted sexual behaviour or advances for transgender autistic adults, however this was limited to people whose sex at birth was female and was compared to cisgender females only. Further research is needed to understand the association between gender and violence amongst autistic adults.

Overall, the limited research to date indicates that autistic people may be at heightened risk for victimisation and violence in adulthood, particularly sexual violence and especially for autistic women. However, the full extent and nature of violence exposure during adulthood is not yet fully understood. Most studies have focused on sexual victimisation, with little known about the rates of physical and other forms of violence (e.g., stalking, sexual harassment). Furthermore, all of these forms of violence rarely occur in isolation, with many individuals experiencing multiple (more than one type) of violence (Fisher et al., 2016). Multiple victimisation has been shown to be common amongst autistic children and adults with intellectual disabilities (Codina et al., 2022; Pfeffer, 2016) and is associated with more detrimental effects on mental health and more severe psychiatric symptoms (Appleyard et al., 2005; Finkelhor et al., 2007; Turner et al., 2016). Similarly, repeated incidents of the same type of violence are commonly reported by people, including disabled people (Classen et al., 2005; Conley et al., 2017; Nixon et al., 2017). Low rates of reporting violence to authorities by disabled people and poor management of these reports by criminal justice professionals (Child et al., 2011; Codina & Pereda, 2021; Fogden et al., 2016) also contribute to increased risk and trauma (McGilloway et al., 2020. To our knowledge, no existing studies have investigated multiple or repeated forms of victimisation in relation to autistic adults, and nor have they examined reporting or conviction rates.

### The Current Study

The current study investigated the prevalence and gender patterns of violence in a sample of autistic adults without intellectual disability. In so doing, we built on existing research to understand whether autistic adults without co-occurring intellectual disability experience a wider range of violence types and/ or more frequent violence exposure beyond childhood compared to non-autistic adults. We also examined whether the gender patterns for violence that are usually seen in the general population (that is, higher rates of physical violence for men and sexual violence for women) are also evident amongst autistic adults. We hypothesized that, compared to non-autistic adults, autistic adults would report higher rates of violence (sexual harassment, stalking and harassment, sexual violence, physical violence) since the age of 15. We also hypothesized that autistic adults would be more likely to report experiencing multiple forms of violence and repeated instances of the same form of violence than non-autistic adults. We predicted that gender differences in patterns of violence (more physical violence reported by men and more sexual violence reported by women) would be apparent in the non-autistic group, but not in the autistic group.

Finally, we also sought to investigate the contextual factors surrounding incidents of violence experienced by autistic adults, which have thus far been overlooked in existing research. In the typical population, perpetrators of violence against women are mostly male partners and men are more likely to be victimised by strangers (Harrell, 2012a; WHO, 2002). People are more likely to experience violence during early adulthood (Conley et al., 2017; Fedina et al., 2020) and violence often involves drug and alcohol use of one or both of the parties involved (Duke et al., 2018; Espelage et al., 2018). To address this issue, we therefore elicited information about the context of the most recent incidents from autistic adults including perceptions related to the seriousness of the incident at the time, whether the incident was reported to anyone and the outcome of any matters reported to police.

## Methods

### Participants

Two hundred and fifty participants took part in this study, including 122 autistic adults who either self-reported an independent clinical diagnosis of an autism spectrum condition (n = 104) or self-identified as autistic but did not have a professional diagnosis (n = 18), and 128 non-autistic adults. Inclusion criteria included being 19 years or older and the ability to read and write in English. As people with severe mental illness have consistently been found to be at increased risk of violence victimisation (Kamperman et al., 2014; Khalifeh et al., 2016; Teplin et al., 2005) we excluded individuals with a previous diagnosis of severe mental illness (defined as schizophrenia or other delusional disorder or bipolar disorder) in order to minimise any potential confounding effects. None reported a diagnosis of intellectual disability. Eighteen participants in the non-autistic group scored above the cut-off score of 65 (range = 66–81) on the Autism Quotient Short Form (AQ-S; Hoekstra et al., 2011), indicative of an autism spectrum condition, and four of the self-identified autistic group scored below the cut-off score (range = 55–64). These participants were excluded from subsequent analyses. As a professional diagnosis of autism is typically assigned following extensive assessment utilising a range of diagnostic tools, all autistic participants who reported a clinical autism diagnosis were included regardless of AQ-S score (see Table 1 for scores).

**Table 1:** Participant demographics

	Autistic (n = 118) n (%)	Non-autistic (n = 110) n (%)
Age		
Mean	35.79 (SD = 13.33)	37.13 (SD = 12.19)
Range	19–70	19–66
Gender		
Men	25 (21.2%)	25 (22.7%)
Women	77 (65.3%)	84 (76.4%)
Other	15 (12.7%)	1 (0.9%)
Prefer not to say	1 (0.8%)	0
Sexual orientation		
Heterosexual	55 (46.6%)	97 (88.2%)
Gay/Lesbian/Homosexual	12 (10.2%)	6 (5.5%)
Bisexual	13 (11.01%)	6 (5.5%)
Pansexual	9 (7.62%)	0
Asexual	6 (5.08%)	0
Queer	5 (4.23%)	0
Questioning	2 (1.69%)	0
Other (unspecified)	9 (7.62%)	0
Prefer not to say	7 (5.9%)	1 (0.9%)
Country		
Australia	64 (54.2%)	63(57.3%)
United Kingdom	20 (16.9%)	29 (26.4%)
USA	25 (21.2%)	12 (10.9%)
New Zealand	2 (1.7%)	1 (0.9%)
Canada	5 (4.2%)	0
Other	2 (1.7%)	5 (4.5%)
Ethnicity		
White	102 (86.44%)	81 (73.6%)
Asian or Asian Indian	7 (5.93%)	18 (16.4%)
Other	9(7.62%)	11 (10%)
Highest education level		
University	75 (63.6%)	70 (63.7%)
Vocational or Trade certificate	25 (21.2%)	12 (10.9%)
High school	14 (11.9%)	23 (20.9%)
Did not complete High school	3 (2.5%)	3 (2.7%)
Prefer not to say	1 (0.8%)	2 (1.8%)
Currently employed	68 (57.6%)	71 (64.5%)
AQ-S scores		
Mean (SD)	87.78 (SD = 9.58)	56.20 (SD = 8.69)
Range	63–108	30–65
Co-occurring mental health condition	70 (59.3%)	15 (15.5%)
Anxiety disorder	57 (48.3%)	9 (8.2%)
Mood disorder	37 (31.4%)	10 (9.1%)
PTSD	30 (25.4%)	4 (3.6%)

AQ-S scores are derived from the Autism Quotient-Short Form (Hoekstra et al., 2011) with higher scores indicating greater autistic traits

The final sample therefore consisted of 118 autistic adults, including 104 with a clinical diagnosis and 14 self-identified, and 110 non-autistic adults (see Table 1). There was a higher proportion of autistic participants who were of White ethnic background (*Χ*^2^(1, 228) = 5.89, *p* = .015, *φ* = .161) and who identified as gender other than man or woman (*Χ*^2^ (2, 277) = 12.35, *p* = .002, *φ* = − .233) compared to the non-autistic group. The 15 adults who identified as gender other than man or woman included 10 who identified as non-binary, three as gender-fluid, one as intersex, one as transgender male and one did not specify. For the purposes of analyses in this study, we combined these individuals into one group using the umbrella term of transgender and gender-nonconforming (TGNC; APS, 2020). There was no significant difference between the groups in the proportion of men and women (*Χ*^2^ (1, 211) = .07, *p* = .78). A significantly higher proportion of the non-autistic group reported that they were heterosexual compared to the autistic group (*Χ*^2^ (1, 220) = 40.06, *p* < .001, *φ* = − .427). The proportion of autistic adults who reported a co-occurring mental health condition was significantly higher than non-autistic adults (*Χ*^2^(1, 228) = 46.43, *p* < .001). There were no significant group differences in age (*U* = 6170*, z* = − .64*, p* = .52), proportion who were employed (*Χ*^2^(1, 223) = 1.42, *p* = .23) or had completed tertiary qualifications (*Χ*^2^(1, 225) = 3.31, *p* = .07, *φ* = − .121). As expected, the autistic group’s mean AQ-S scores was significantly higher than the non-autistic group (*U* = 90,* z* = − 12.86,* p* < .001, *r* = .85).

### Procedure

Ethical approval was obtained from the Human Research Ethics Committee (approval number 5202059192411) at Macquarie University. Recruitment into the study occurred between February and June 2021. The survey was online and hosted on the Qualtrics platform (https://qualtrics.com) We recruited autistic participants via online flyers distributed by email and social media to autism-specific organisations, Facebook groups and personal networks in Australia and overseas. Snowball sampling methods were also introduced to boost recruitment, with participants asked to share the researcher’s contact details via their networks. We recruited non-autistic participants via advertisements to undergraduate psychology student, Facebook groups and personal networks. Students accessed the survey via an online platform hosted by the university which advertises research participation opportunities for students for course credit. An additional 34 non-autistic adults were recruited via a crowd-sourcing platform for online research (https://prolific.co). All participants provided consent to take part prior to commencement of the survey. Given the online nature of the study, we employed several strategies to protect the quality of the data collected. Individuals recruited via social media were required to contact the first author via email in order to obtain an individual link to the survey. The first author also reviewed the speed of survey completion. All surveys completed in under 6 min were discarded, based on a calculation of two standard deviations below the mean completion time of approximately 10 min as recommended by Teitcher et al. (2015). The survey included a CAPTCHA code and attention check and/or “trap” questions i.e. spell the word “WORLD” backwards, provide postcode and year of birth at the end of the survey which was compared to IP address location and age provided at the beginning of the survey. We discarded nine surveys due to inconsistencies detected.

### Measures

#### Demographics and Diagnostic History

We asked participants to report demographic characteristics including age, gender, sexual orientation, ethnicity, education level, employment status and relationship status along with information related to prior diagnoses of any autism, ADHD and mental health conditions.

#### Experiences of Violence

We gathered information about the nature and extent of violence since the age of 15 using categories and descriptions derived from the Australian Bureau of Statistics (ABS) Personal Safety Survey (ABS, 2016). Participants were asked to indicate using a “yes/no” format whether they had experienced any of the following forms of violence:

- *sexual harassment,* including someone exposing himself/herself to them, making them uncomfortable by making inappropriate comments about their body or their sex life or sending them indecent texts or emails;
- *stalking and harassment,* including someone following them or watching them, interfering with or damaging their property, loitering or hanging around their home, workplace or where they socialise, hacking their online account or social media without their consent;
- *sexual violence,* anyone including relationship partners, ever forcing them or trying to force them into sexual activity against their will; and
- *physical violence,* including anyone pushing, shoving, hitting, kicking, throwing things at them or attacking them with an object.

Participants who indicated that they had experienced a form of violence were asked to indicate how many times this had occurred since the age of 15; that is, once, twice, 3–5 times or more than 5 times.

We also collected additional information relating to the most recent incident of physical and sexual violence from autistic adults, including age range at time of incident (under 18, 18 to 24, 25 to 34, 35 or over), gender of perpetrator, relationship to perpetrator, whether they were under the influence of drugs or alcohol, whether they perceived the incident to be a criminal act at the time, whether they confided in others and/or reported to police, any reasons for not reporting, and whether there were any convictions arising (see Online Appendix for full version of Experiences of Violence measure). Time taken to complete the measure ranges from approximately 1 min to 6 min.

#### Screening for Inclusion

The Autism Quotient Short Form (AQ-S) is an abridged version of the 50-item Autism Quotient (AQ) (Baron-Cohen et al., 2001) and is a screening instrument for autistic traits (Hoekstra et al., 2011). Each of its 28 items (e.g., ‘I prefer to do things the same way over and over again’; ‘I usually notice car number plates or similar strings of information’; ‘I find social situations easy’) are rated on a 4-point scale ranging from 1 (*definitely agree*) to 4 (*definitely disagree*). Higher scores indicate higher autistic traits. High Pearson’s correlations (r = 0.93–0.95) have been reported between the full AQ and AQ-S (Hoekstra et al., 2011). Kuenssberg et al. (2014) confirmed good fit of the AQ-S structure in a sample of autistic individuals without intellectual disability, whereby total AQ-S mean scores yielded good internal consistency (Cronbach’s α of 0.84). In this sample, internal consistency ranged from good to excellent with a Cronbach alpha of .94 for the full sample, .85 for the non-autistic group and .79 for the autistic group. Excellent test accuracy (Area Under the Curve = .97) for distinguishing latent traits in autistic and non-autistic participants has been demonstrated (Hoekstra et al., 2011).

### Data Analysis

All data analysis was conducted using SPSS Statistics Version 25. Preliminary tests of normality were conducted using the Kolmogorov–Smirnov statistic; subsequent analysis was determined by the normality results for each variable. To begin, we used Chi-square analysis and Mann Whitney *U* tests to assess group differences on demographics, followed by the rates of different forms of violence—sexual harassment, stalking and harassment, sexual violence and physical violence—and chi square analysis to determine whether violence rates differed across autistic and non-autistic adults or by gender. Next, we created two additional dependent variables: (1) *Multiple violence* (more than one form of violence reported) and (2) *Repeated violence* (three or more instances of any one form of violence). We examined the rates of multiple and repeated violence and used chi square analysis to determine group differences on these variables. As our final research question related to the nature and context of violence for autistic adults, descriptive statistics were reported in relation to the most recent incidents of violence for the autistic group only.

### Community Involvement Statement

This project received input from an advisory group consisting of three autistic adults, who were paid for their time and expertise. The researchers met with them twice over the course of the study, including to gain their opinions on the research questions and review the questionnaire items to check for accessibility and acceptability. In response to their feedback, one question pertaining to police reporting was modified to include examples of possible reasons for not reporting to police and one question was added to cover the possibility that incidents may be reported to police by a friend or family member on someone’s behalf rather than by the person themselves. The advisory group also participated in a group meeting where the methods and results were summarised and the interpretation of the findings were discussed. They were subsequently provided with a copy of the manuscript and invited to make any additional comments on the authors’ interpretations. All three members of the advisory group advised that the final manuscript was consistent with the input and interpretations they had previously provided and no changes were requested.

## Results

### Rates of Reported Violence for Autistic and Non-autistic Adults

As expected, a significantly higher proportion of autistic adults reported experiencing each form of violence compared to non-autistic adults (Table 2). Just over three-quarters of the autistic group (75.4%) reported sexual harassment compared to approximately half of the non-autistic group (56.4%) (*χ*^*2*^*(*1, 225) = 10.02, *p* = .002, *φ* = − .211). Similarly, a significantly higher proportion of autistic adults (58.5%) reported stalking and harassment compared to non-autistic adults (27.3%) (*χ*^*2*^*(*1, 226) = 22.68, *p* < .001, *φ* = − 0.317). Almost 60% of autistic adults reported experiencing sexual violence (56.8%) and physical violence (58.5%). These rates were significantly higher than those reported by non-autistic adults (28.2% for sexual violence: *χ*^2^ (1, 221) = 22.04, p < .001, φ = − .316; 36.4% for physical violence; *χ*^2^ (1, 226) = 11.21, *p* = .001, *φ* = − .223). Approximately three quarters of Autistic adults (76.3%) reported multiple types of violence compared to approximately half (46.4%) of the non-autistic group (*χ*^2^ (1, 220) = 24.43, *p* < .001, *φ* = .333). A significantly higher proportion of autistic adults (76.3%) also indicated that they had experienced repeated instances of at least one form of violence compared to non-autistic adults (54.5%) (*χ*^2^ (1, 228) = 11.94, *p* = .001, *φ* = 229).

**Table 2:** Comparison of rates of violence reported by Autistic and non-autistic adults

Type of violence	Autistic adults n = 118	Non-autistic adults n = 110
	n (%)	n (%)
Sexual harassment	89 (75.4%)	62 (56.4%)
Stalking and harassment	69 (58.5%)	30 (27.3%)
Sexual violence	67 (56.8%)	31 (28.2%)
Physical violence	69 (58.5%)	40 (36.4%)
Multiple (more than one type) violence	90 (76.3%)	51 (46.4%)
Repeated violence (three or more experiences of any form of violence)	90 (76.3%)	60 (54.5%)

Participants were able to select all options that applied to them therefore percentages do not add up to 100

### Gender Patterns in Rates of Reported Violence

#### Rates of Violence Reported by Autistic and Non-autistic Participants by Gender

There was no significant difference in rates of stalking and harassment or physical violence for autistic and non-autistic men (stalking and harassment: *χ*^*2*^ (1, 49) = .98, *p* = .322; physical violence, *χ*^*2*^* (*1, 49) = .23, *p* = .628) (see Table 3). Similarly, the proportion of autistic men who reported experiencing multiple types of violence or repeated instances of violence was not significantly different to non-autistic men (multiple violence, *χ*^*2*^* (*1, 48) = 3.18, *p* = .074; repeated violence, *χ*^*2*^* (*1, 50) = .347, *p* = .556). Few participants in the non-autistic group reported sexual harassment and sexual violence, which meant that statistical analysis could not be conducted.

**Table 3:** Comparison of rates of violence reported by autistic and non-autistic participants by gender

Type of violence	Autistic men (n = 25)	Non-autistic men (n = 25)	Between groups comparison
	n (%)	n (%)	
Sexual harassment	14 (56%)	4 (16%)	
Stalking and harassment	12 (48%)	9 (36%)	*χ*^*2*^ (1, 49) = .98, *p* = .322
Sexual violence	10 (40%)	2 (8%)	
Physical violence	16 (64%)	15 (60%)	*χ*^*2*^ (1, 49) = .23, *p* = .628
Multiple (more than one type) violence	16 (64%)	11 (44%)	*χ*^*2*^ (1, 48) = 3.18, *p* = .074
Repeat violence	17 (68%)	15 (60%)	*χ*^*2*^ (1, 50) = .347, *p* = .556

	Autistic women n = 77	Non-autistic women n = 84	
	n (%)	n (%)	
Sexual harassment	61 (79.2%)	57 (67.9%)	*χ*^*2*^ (1, 158) = 3.34, *p* = .068
Stalking and harassment	47 (61.0%)	20 (23.8%)	*χ*^*2*^ (1, 160) = 22.4, *p* < .001 *φ* = .374
Sexual violence	47 (61.0%)	29 (34.5%)	*χ*^*2*^ (1, 157) = 12.79, *p* < .001 *φ* = .285
Physical violence	42 (54.5%)	24 (28.6%)	*χ*^*2*^ (1, 160) = 10.83, *p* = .001 *φ* = .26
Multiple violence	60 (77.9%)	39 (46.4%)	*χ*^*2*^ (1, 155) = 18.17, *p* < .001 *φ* = .342
Repeat violence	58 (75.3%)	44 (52.4%)	*χ*^*2*^ (1, 161) = 9.11, *p* = .003 *φ* = .238

	Autistic TGNC n = 15	Non-autistic TGNC n = 1
	n (%)	n (%)
Sexual harassment	13 (86.7%)	*
Stalking and harassment	9 (60.0%)	*
Sexual violence	9 (60.0%)	*
Physical violence	10 (66.7%)	*
Multiple (more than one type) violence	13 (86.7%)	*
Repeat violence	14 (93.3%)	*

*Not reported to protect subgroups with fewer than five participants. Statistical analyses for sexual harassment and sexual violence for men were not conducted due to low case numbers. Participants were able to select all options that applied to them therefore percentages do not add up to 100

In contrast, and strikingly, a significantly higher proportion of autistic women reported stalking and harassment, sexual violence and physical violence compared to non-autistic women. Sixty-one percent of autistic women reported stalking and harassment compared to 23.8% of non-autistic women (*χ*^*2*^* (*1, 160) = 22.4, *p* < .001 *φ* = .374). Just over 60% of autistic women (61%) reported sexual violence compared to about one-third (34.5%) of non-autistic women (*χ*^*2*^* (*1, 157) = 12.79, *p* < .001 *φ* = .285). Approximately half (54.5%) of autistic women reported physical violence compared to 28.6% of non-autistic women (*χ*^*2*^* (*1, 160) = 10.83, *p* = .001 *φ* = .26). In addition, the proportions of autistic women who reported experiencing multiple types, and repeated instances, of violence were significantly higher than non-autistic women. The majority of autistic women (77.9%) reported multiple violence compared to 46.4% of non-autistic women (*χ*^*2*^* (*1, 155) = 18.17, *p* < .001 *φ* = .342). Approximately three-quarters of autistic women (75.3%) reported repeated instances of violence compared to just over half of the women in the non-autistic group (52.4%) (*χ*^*2*^* (*1, 161) = 9.11, *p* = .003 *φ* = .238). There was, however, no significant difference in reports of sexual harassment (*χ*^*2*^* (*1, 158) = 3.34, *p* = .068) (Table 3).

Due to the low number of TGNC non-autistic adults (n = 1), it was not possible to compare rates of violence across groups. However, a high proportion of TGNC autistic adults reported experiencing each of the four types of violence (ranging from 60% reporting stalking and harassment to 86.7% reporting sexual harassment). Approximately 86% reported experiencing more than one type of violence and 93% reported repeated instances of at least one form of violence. These proportions are comparable to those reported by autistic women (see Table 3).

#### Patterns of Violence by Gender Within Each Group (Autistic, Non-autistic)

Table 4 shows the results of between-groups analysis of the pattern of violence by gender for both autistic and non-autistic adults. For autistic adults, as predicted, there were no significant differences in rates of reported violence between men and women or between men, women and TGNC people for sexual violence or physical violence. For non-autistic adults, the typical pattern of higher rates of physical violence reported by men compared to women was borne out (60.0% for men compared to 28.9% for women; see also Table 3). Analysis for sexual violence could not be conducted due to low numbers in the male non-autistic group (see Table 3).

**Table 4:** Patterns of violence by gender within each group (Autistic, non-Autistic) separately

	Between groups comparisons men and women (Autistic)	Between groups comparisons (men, women and TGNC) (Autistic)	Between groups comparisons men and women (non-autistic)
Sexual violence	*χ*^*2*^ (1, 98) = 3.55, *p* = .059	*χ*^*2*^ (2, 111) = 4.17, *p* = .124	n/a
Physical violence	*χ*^*2*^ (1, 101) = 1.1, *p* = .294	*χ*^*2*^ (2, 116) = 1.57, *p* = .457	*χ*^*2*^ (1,108) = 8.05, *p* = .005, *φ* = − .273

Statistical analyses of gender patterns for sexual violence was not conducted for non-Autistic adults due to low case numbers

### Recent Incidents of Violence for Autistic Adults

Autistic adults provided details about their most recent incident of sexual harassment, stalking and harassment, sexual violence and physical violence (294 incidents in total).

#### Context of Most Recent Incidents

The context surrounding the most recent incidents (age, gender and relationship to perpetrator, whether the incident was reported to anyone or drugs or alcohol were involved) are described separately for autistic men, women and TGNC adults (Table 5).

**Table 5:** Context of most recent violence reported by autistic men, women and TGNC adults

	Sexual harassment n %			Stalking and harassment n %			Sexual violence n %			Physical violence n %		
	Men	Women	TGNC	Men	Women	TGNC	Men	Women	TGNC	Men	Women	TGNC
	n = 14	n = 61	n = 13	n = 12	n = 47	n = 9	n = 10	n = 47	n = 9	n = 16	n = 42	n = 10
Age												
25 or over	6	36	9	12	20	4	4	28	7	7	22	7
	42.9%	59%	60%	100%	42.6%	44.4%	40%	59.6%	77.8%	43.8%	52.4%	70%
Relationship to perpetrator												
Partner	4	12	2	4	19	2	5	24	3	3	18	4
	28.6%	19.7%	15.4%	33.3%	40.4%	22.2%	50%	51.1%	33.3%	18.8%	42.9%	40%
Friend/acquaintance	2	17	1	5	10	5	2	13	3	8	2	1
	14.2%	27.9%	7.7%	41.8%	21.3%	55.5%	20%	27.6%	33.3%	50%	4.8%	10%
Stranger	3	24	5	1	9	1	0	5	2	2	3	2
	21.4%	39.4%	38.5%	8.3%	19.1%	11.1%		10.6%	22.2%	12.5%	7.1%	20%
Work colleague	3	6	2	1	3	0	2	0	1	0	1	0
	21.4%	9.8%	15.4%	8.3%	6.4%		20%		11.1%		2.4%	
Client/customer	1	1	2	0	2	0	1	0	0	1	1	2
	7.1%	1.6%	15.4%		4.3%		10%			6.2%	2.4%	20%
Family member/relative	1	0	1	1	4	1	0	1	0	2	11	1
	7.1%		7.7%	8.3%	9.5%	11.1%		2.1%		12.5%	26.2%	10%
First date	0	0	0	0	0	0	0	4	0	0	0	0
								8.6%				
Police officer	0	0	0	0	0	0	0	0	0	0	2	0
											4.8%	
Prefer not say	0	1	0	0	0	0	0	0	0	0	0	0
		1.6%										
Gender of perpetrator												
Male	3	56	12	6	42	7	3	43	8	9	33	10
	21.4%	91.8%	92.3%	50%	89.4%	77.8%	30%	91.5%	88.9%	56.3%	78.6%	100%
Female	11	5	1	6	5	1	7	4	1	7	9	0
	78.6%	8.2%	7.7%	50%	10.6%	11.1%,	70%	8.5%	11.1%	43.8%	21.4%	
Confided in someone	4	29	7	9	39	8	4	13	6	9	21	4
	28.6%	47.5%	53.8%	75%	83%	88.9%	40%	27.7%	66.7%	56.3%	50%	40%
Under influence	n/a	n/a	n/a	n/a	n/a	n/a	1	10	2	2	4	1
							10%	21.3%	22.2%	12.5%	9.5%	10%

##### Autistic Men

Twenty-two autistic men described 52 of their most recent incidents of violence: 14 instances of sexual harassment, 12 stalking and harassment incidents, nine instances of sexual violence and 16 of physical violence (Table 5). Approximately 40% of sexual harassment (n = 6, 42.9%), sexual violence (n = 4, 40%) and physical violence incidents (n = 7, 43.8%) and all of the most recent stalking and harassment incidents occurred at age 25 or over. Partners or ex-partners were the most commonly reported perpetrators of sexual harassment and sexual violence (sexual harassment, n = 4, 28.6%; sexual violence, n = 5, 50%). For stalking and harassment and physical violence the most common perpetrator was a friend or acquaintance (stalking and harassment, n = 5, 41.8%; physical violence, n = 8, 50%). Strangers accounted for 21.4% of sexual harassment incidents (n = 3) and 12.5% of physical violence (n = 2). In terms of gender of the perpetrator, the majority of sexual harassment and sexual violence incidents were perpetrated by women (sexual harassment, n = 11, 78.6%; sexual violence, n = 7, 70%). For stalking and harassment, the gender of the perpetrator was evenly split between men and women, that is, six incidents each. In the case of physical violence, nine incidents involved a male perpetrator (56.3%) and seven incidents involved a female perpetrator (43.8%). Following 75% of stalking incidents (n = 9) and 56% of physical violence incidents (n = 9), Autistic men reported that they confided in someone about their experience. The proportions were lower for sexual harassment (n = 4, 28.6%) and sexual violence (n = 4, 40%). There was only one incident of sexual violence and two incidents of physical violence where the autistic man reported that they were under the influence of drugs or alcohol.

##### Autistic Women

Sixty-eight autistic women described 197 of their most recent incidents of violence: 61 instances of sexual harassment, 47 stalking and harassment incidents, 47 instances of sexual violence and 42 of physical violence (see also Table 5). Approximately 60% of sexual harassment and sexual violence incidents and half of the physical violence incidents occurred at age 25 or over (sexual harassment, n = 35, 59%; sexual violence, n = 28, 59.6%; physical violence, n = 22, 52.4%). Strangers were the most frequently nominated perpetrator type for sexual harassment incidents (n = 24, 39.4%). Other perpetrators of sexual harassment were friends and acquaintances (n = 17, 27.9%), partners or ex-partners (n = 2, 19.7%) and work colleagues (n = 6, 9.8%). Partners or ex-partners were the most commonly reported perpetrators of stalking and harassment (n = 19, 40.4%), sexual violence (n = 24, 51.1%) and physical violence (n = 18, 42.9%). About one-quarter of the physical violence incidents reported by autistic women were perpetrated by family members (n = 11, 26.2%). Friends or acquaintances were responsible for 21.3% of stalking and harassment incidents (n = 10) and 27.6% of sexual violence incidents (n = 13). In terms of gender of the perpetrator, the vast majority of violence incidents were perpetrated by men (sexual harassment, n = 56, 91.8%; stalking and harassment, n = 42, 89.4%; sexual violence, n = 43, 91.5%; physical violence, n = 3, 78.6%). Women were most likely to confide in someone about stalking and harassment (n = 39, 83%) and least likely to confide about sexual violence (n = 13, 27.7%). In about half of the cases of stalking and harassment (n = 29, 47.8%) and physical violence (n = 21, 50%), autistic women reported that they confided in someone about their experience. Ten incidents of sexual violence (21.3%) and four incidents of physical violence (9.5%) involved drugs or alcohol.

##### Autistic TGNC

Fifteen autistic TGNC adults described 41 of their most recent incidents of violence: 13 instances of sexual harassment, nine stalking and harassment incidents, nine instances of sexual violence and 10 of physical violence (Table 5). The majority of sexual harassment, sexual violence and physical violence incidents occurred at age 25 or over (sexual harassment, n = 9, 60%; sexual violence, n = 7, 77.8%; physical violence, n = 7, 70%). The most common perpetrator of sexual harassment was reported to be a stranger (n = 5, 38.5%). Just over half of the stalking and harassment incidents (n = 5, 55.5%) involved friends or acquaintances. Physical violence was most commonly perpetrated by a partner or ex-partner (n = 4, 40%). Three cases each (33.3%) of sexual violence incidents involved partners or ex-partners or friends and acquaintances. In terms of gender of the perpetrator, the majority of violence incidents were perpetrated by men (sexual harassment, n = 12, 92.3%; stalking and harassment, n = 7, 77.8%; sexual violence, n = 8, 88.9%; physical violence, n = 10, 100%). They were most likely to confide in someone about stalking and harassment (n = 8, 88.9%) and least likely to confide about physical violence (n = 4, 40%). In about half of the cases of stalking and harassment (n = 7, 53.8%) and two-thirds of sexual violence (n = 6, 66.7%), TGNC autistic adults reported that they confided in someone about their experience. Two incidents of sexual violence (22.2%) and only one incident of physical violence (10%) involved drugs or alcohol.

### Perceptions About Violence and Reporting to Police

For each of the 294 most recent incidents of violence, autistic adults were asked if they viewed the incident as a crime, whether they reported the incident to police and whether anyone was convicted of a crime in relation to the incident. One quarter of the incidents (n = 75, 25.5%) were viewed as crimes at the time. Forty-one were reported to police (13.9%) and, of these, five resulted in a conviction (see Table 6).

**Table 6:** Perceptions of incident as a crime, reporting to police and conviction rates for most recent incidents of violence as reported by autistic adults

	Sexual harassment	Stalking and harassment	Sexual Violence	Physical violence	Total
	n = 89	n = 69	n = 67	n = 69	n = 294
	n (%)	n (%)	n (%)	n (%)	n (%)
Viewed as a crime at the time	14 (15.7%)	24 (34.8%)	16 (23.9%)	21 (30.4%)	75 (25.5%)
Reported to police	4 (4.5%)	16 (23.2%)	7 (10.4%)	14 ((20.3%)	41 (13.9%)
Resulted in conviction	0	3 (4.3%)	1 (1.5%)	1 (1.4%)	5 (1.7%)

When autistic participants viewed what had happened as a crime but did not report to police (n = 34), the most common reason provided (n = 28, 82.3%) was that they did not think there was anything police could do to help them. In about two-thirds of cases (n = 22, 64.7%) they indicated that they did not trust police. For approximately 60% of incidents, they reported a fear of legal processes (n = 21, 61.7%) and a perception that they would not be believed (n = 20, 58.2%). In 55% of incidents (n = 19), the autistic person did not report the incident because they feared repercussions from the person responsible. Feelings of shame and embarrassment (n = 14, 41.7%) and communication difficulties (n = 12, 35.3%) were endorsed as other reasons for not reporting to police.

## Discussion

The aim of the current study was to examine the nature and extent of violence exposure among autistic people without intellectual disability during adulthood. To our knowledge, this is the first study to measure both multiple and repeated victimisation across a broad range of violence types. In line with our hypotheses, we found that autistic adults reported higher rates of a range of violence types and were more likely to experience multiple forms of violence and repeated instances of the same type of violence than non-autistic adults. Autistic adults reported higher rates of sexual harassment, stalking and harassment, sexual violence and physical violence than non-autistic adults. In addition, approximately three-quarters of autistic adults had experienced two or more forms of violence and repeated instances of the same violence type since the age of 15 compared to approximately half of the non-autistic adults. These findings are consistent with the limited research that has been conducted in this area (Griffiths et al., 2019; Weiss & Fardella, 2018) and highlight that the risk of increased violence is wide-ranging, extends beyond childhood and is not limited to autistic people with co-occurring intellectual disability.

Our finding that autistic adults are not only more likely to report having experienced various forms of violence, but are also more likely to report multiple and repeated violence is particularly concerning given the elevated risk of significant mental health impacts associated with multiple victimisation (Appleyard et al., 2005; Finkelhor et al., 2007; Turner et al., 2016). The phenomenon of repeat victimisation, that is, that victimisation occurs disproportionately among certain individuals is a consistent finding amongst the general population (Farrell et al., 1995; Tseloni & Pease, 2003; Pease, 1998), with numerous studies finding that prior victimisation strongly predicts future victimisation (Finkelhor et al., 2007; Menard, 2002; Outlaw et al., 2002). A clear theoretical explanation for why this is the case, however, has yet to be determined. One explanation may be that risk factors for violence are relatively stable, for example, lifestyle patterns that may place people in dangerous situations such as a person’s tendency to participate in ‘high risk’ activities or continuing involvement in conflictual relationships (Dean et al., 2007). Alternatively, early experiences of victimisation may affect an individual’s personality, social development and relationship patterns, which may in turn lead to interpersonal problems that increase vulnerability (Classen et al., 2005; Goodman et al., 2001). The limited research with autistic adults has tended to focus on the relationship between individual characteristics and violence exposure with the rationale being that social communication difficulties may increase vulnerability to manipulation or coercion by others and impede an autistic person’s ability to identify and remove themselves from risky situations. Autistic people’s difficulty generalising and learning from prior experiences (Cannon et al., 2021; Pellicano & Burr, 2012) may also be a contributing factor to repeat victimisation among this group. Nevertheless, a clear association between individual characteristics, including social competence, emotion regulation difficulties and autistic traits, has yet to be demonstrated (Gibbs et al., 2021; Roberts et al., 2015; Weiss & Fardella, 2018).

Bronfenbrenner’s (1977) ecological theory when applied to understanding violence victimisation considers individual characteristics, however the emphasis is on the interplay of these characteristics within the social contexts in which people are embedded. The negative attitudes, stigma and stereotyping of autistic people (Botha & Frost, 2020; Cage et al., 2019; Sasson et al., 2017; Shtayermman, 2009) may contribute to potential perpetrators viewing an autistic person as inferior, incapable and compliant and therefore as an “attractive victim” (Meer & Combrinck, 2015). In addition, the social isolation that many autistic adults experience (Howlin, 2013) may limit access to important social supports and networks and increase the likelihood of them remaining in unhealthy or even abusive relationships. Autistic children experience high rates of bullying and peer victimisation (Griffiths et al., 2019; Pfeffer, 2016) and have also been shown to be more likely to experience abuse and maltreatment (Dinkler et al., 2017; Mandell et al., 2005). This early exposure to violence may result in psychological and emotional impacts such as poor self-concept, feelings of powerless and betrayal and post-traumatic symptoms which may in turn exacerbate vulnerability to repeat victimisation. In addition, autistic people have been found to be less likely to confide in others about their experiences of physical and sexual violence (Gibbs et al., 2021). Without support from family, professionals or services autistic people may have difficulty developing protective behaviours and/or extricating themselves from dangerous situations contributing to high rates of multiple and repeat victimisation. Future research must therefore examine individual, contextual and societal aspects that may contribute to elevated risk for autistic people. Understanding characteristics that increase vulnerability may assist clinicians to identify individuals who are at particular risk and inform the development of targeted education strategies and support services. Ultimately, however, research and policymaking should target the broader contextual and societal factors that place autistic people at increased risk for violence so that such risk can be mitigated.

Since gender is often associated with different types of violence, we compared rates of violence for autistic and non-autistic adults separately for men and women. Autistic women reported higher rates of all forms of violence. In contrast, there was no difference in rates of stalking and harassment, physical violence, multiple violence or repeated violence for autistic and non-autistic men. The number of non-autistic men who reported sexual harassment and sexual violence was too low to conduct statistical comparison. However, the proportions of autistic men who reported sexual harassment and sexual violence are considerably higher than prevalence rates from large population samples of violence during adulthood (56% for sexual harassment and 40% for sexual violence in our sample compared to 25% for sexual harassment and 4.7% for sexual violence from the Australian Personal Safety Survey; ABS, 2016). High rates of sexual victimisation have been found in studies of men with intellectual disability (Platt et al., 2017; Saxton et al., 2006). Disabled men, including autistic men, may be less likely to behave in ways that conform to masculinity stereotypes (Powers et al., 2008) decreasing their power in relationships and leaving them vulnerable to the abuses and subjugation typically experienced by women (Shakespeare, 1999).

The small sample size of TGNC autistic adults and the lack of a comparison group meant that we were unable to conduct any comparative analysis. Nevertheless, rates of reported violence for this group were high across all violence types (ranging from 60% to 86.7%) with an alarmingly high proportion (93.3%) reporting repeat victimisation. This is consistent with research of TGNC people in the general population who have been found to have elevated rates of violence and victimisation compared to cisgender people (Aparicio-Garcia et al., 2018; Grant et al., 2011; James et al., 2016). These gender-related findings can be interpreted within an intersectionality framework (Crenshaw, 1989), which posits that multiply marginalised identities intersect to amplify the risk of violence exposure. Being an autistic person represents one social identity that confers additional risk (a person with a disability). Being an autistic woman or TGNC person combines the added risk of disability with the risk associated with being female or of non-conforming gender.

We also analysed rates of reported violence by gender for both autistic and non-autistic groups separately. As expected, gender differences in the patterns of violence (more physical violence reported by men and more sexual violence reported by women) were apparent in the non-autistic but not the autistic group. This pattern of narrowing of gender differences has also been found amongst people with intellectual disability and those with severe mental illness (Cohen et al., 2006; Harrell, 2012b; Dean et al., 2007; de Mooji et al., 2015; Smith, 2008) and in our study is due to the higher rates of sexual violence reported by autistic men and physical violence reported by autistic women. Overall, these findings underscore the importance of reconsidering issues of gender and violence as they apply to autistic people when undertaking research in this area and also when working with autistic people in clinical settings. Caution is warranted, however, given the small sample sizes, particularly for men and TGNC individuals. Further research with larger samples of men, women and TGNC people will provide important information about the nuanced nature of violence across gender for autistic people. In the meantime, clinicians and other service providers need to be aware of the disproportionate rates of victimisation for this population across all types of violence regardless of gender.

To our knowledge, this is the first time that contextual factors for a range of violence types have been investigated for autistic adults. We gathered information about the age range of the individual at the time of the most recent incident, the gender of the perpetrator and their relationship to them, whether drugs and alcohol played a role and whether they had confided in anyone or reported to police. In the general population, violence is most common during the early adulthood period (Conley et al., 2017; Fedina et al., 2020), however a substantial proportion of the most recent incidents reported by autistic adults occurred after age 25, ranging from approximately 42% of recent sexual harassment incidents for men and stalking/harassment incidents for women, up to 100% of the stalking and harassment incidents reported by autistic men. Just over three quarters of the most recent sexual violence incidents reported by TGNC autistic adults occurred after age 25. Although the mechanisms that result in disproportionately high rates of violence victimisation amongst autistic adults are yet to be understood, these findings indicate that certain contextual risk factors such as risky sexual behaviours and substance use (Duval et al., 2020) that predominate in the early adulthood period and correlate with rates of violence in the general population may not be associated with violence for autistic people. In fact, in our study, 80 to 90% of recent incidents of violence did not involve drugs or alcohol which is consistent with the low rates of drug use and alcohol consumption that has been reported in large studies of autistic adults (Weir et al., 2021).

The perpetrators of violence were most commonly known to the autistic person with a substantial proportion of incidents occurring in the context of intimate relationships or friendships. This is consistent with what has been found in the general population for women (ABS, 2016; James et al, 2016). That said, men are most frequently physically assaulted by strangers, which was not the case amongst our sample of autistic men. Similarly, the vast majority of violence perpetrated against men in the general population is by other men, particularly physical violence; however, here, just over 40% of recent physical violence incidents were perpetrated by women. Although we were only able to gather information from a small sample of autistic men, these findings provide further support for the notion that the gender patterns related to violence in the general population may not apply to autistic people and further supports the call for more research in this area. In terms of confiding in others about their experiences of violence, autistics adult reported that they had told someone following the majority of stalking and harassment incidents (75–88.9%). But this was not the case for physical violence, and even less so for sexual violence. These low rates of confiding in others are largely consistent with findings from an earlier study with a different sample (Gibbs et al., 2021) and are deeply concerning. Social support in relation to traumatic experiences and access to appropriate psychological services, both of which have been shown to positively impact well-being after trauma (Dworkin et al., 2018; Rusch et al., 2015) are reliant on the person disclosing violence to their families, networks and/or treating professionals. It is critical that autistic people are able to disclose violence experiences so that appropriate mental health and social supports can be put in place and protective strategies can be identified and implemented to reduce the risk of repeated victimisation.

Approximately one quarter of the recent incidents reported (n = 75) were viewed as a crime at the time by the person involved. This could indicate that the remaining incidents were less serious and did not meet the threshold of criminal violence. The question posed, however, related to the autistic person’s perception of the incident at the time it occurred. It is also possible that even serious incidents of violence were not recognised as such at the time, which is common in the case of intimate partner and dating violence (Mouzos & Makkai, 2004). Regardless, despite considering the incident to be a criminal act only 54% (n = 41) were reported to police, and of those only 5 resulted in a conviction. Low rates of reporting to police and low rates of conviction have also been observed in a number of population groups including college students, individuals with intellectual and other disabilities and TGNC people and is often linked to distrust and fear of the legal system and legal processes (Conley et al., 2017; Donovan & Hester, 2008; McGilloway et al., 2020). The most common reasons for not reporting to police provided by our autistic participants were that police would not be able to assist them, lack of trust in police, fear of legal processes and a perception that they would not be believed. This is consistent with several recent studies investigating interactions between autistic people and police that have described low rates of satisfaction and feelings of fear and mistrust of the justice system and law enforcement (Gibbs & Haas, 2020; Hartmann et al., 2019; Koffer Miller et al., 2021). Efforts to improve reporting and conviction rates should be undertaken so that autistic people feel confident reporting crimes to police, perpetrators are brought to justice and necessary supports and compensation can be provided.

This study has a number of limitations. First, the autistic group was predominantly comprised of those who had completed tertiary study and may not be representative of the broader population of autistic people. We also did not conduct any verification of diagnostic status beyond a self-report screening tool. Second, the sample size of males and TGNC adults was low and there was only one TGNC person in the non-autistic group which meant that no comparative analyses were able to be conducted. Nevertheless, most of the research into autistic people’s experiences of violence in adulthood has been focused on autistic women. This work therefore provides initial insight into the violence experiences of men and TGNC autistic adults. Third, the data were based on retrospective reporting which may have resulted in recall biases. Finally, although we investigated a broader range of violence types than in previous studies, there are other forms of violence and victimisation that were not measured such as emotional and psychological abuse and workplace bullying. Future research aimed at extending knowledge in relation to the full extent and nature of violence experiences should also include these additional forms of violence.

The findings from this study provide further evidence of the extent of violence exposure for autistic people that occurs well into the adult years, with autistic women and those who are transgender or gender nonconforming appearing to be at greatest risk. The study also offers an initial insight into the context of violence experiences among this group with some indication that most violence is perpetrated by men, by people that are known to them, rarely involves drugs or alcohol and often are not reported, even when considered to be a criminal act. These results have several important implications for practice and policy. Health care and allied health practitioners should be made aware of the higher risk of violence for this group so that trauma symptoms are identified and appropriate treatment can be provided. Similarly, specialised training for law enforcement and legal professionals in relation to autism and availability of advocates to support autistic people throughout the legal process may improve criminal justice interactions and outcomes and increase willingness amongst autistic people to report criminal violence to police. The focus of future research should be on gaining a fuller understanding of factors underlying the increased risk of violence amongst autistic people so that targeted prevention strategies can be developed and evaluated. Until such time as any broader societal and systemic factors can be identified and addressed, autistic adolescents and adults should be provided with direct education about their rights, the meaning of consent, what constitutes violence and how to access related information, services and support should they be victimised or have concerns about situations in which they may find themselves.

## Supplementary Information

Below is the link to the electronic supplementary material.

Supplementary file1 (DOCX 18 kb)
