# Supplementary material for: The Extent and Nature of Autistic People’s Violence Experiences During Adulthood: A Cross-sectional Study of Victimisation
Source: J Autism Dev Disord. 2022 Jul 11;53(9):3509–24. doi: 10.1007/s10803-022-05647-3 (PMC10465381; doi:10.1007/s10803-022-05647-3)
Supplement: Supplementary file 1 — Supplementary file1 (DOCX 18 kb) [file 10803_2022_5647_MOESM1_ESM.docx]

APPENDIX – EXPERIENCES OF VIOLENCE MEASURE

1. Since the age of 15, has anyone ever sexually harassed you? (Examples of sexual harassment include someone exposing himself/herself to you, making you uncomfortable by making inappropriate comments about your body or your sex life or sending you indecent texts or emails).

Yes / No / Prefer not to answer

1. Other than sexual harassment, since the age of 15, has anyone ever stalked or harassed you? Examples of stalking/harassment other than sexual harassment include following you or watching you, interfering with or damaging your property, loitering or hanging around your home, workplace or where you socialise, hacking your online account or social media without your consent.

Yes / No / Prefer not to answer

1. Since the age of 15, has anyone, including any relationship partners, ever forced you or tried to force you into sexual activity against your will?

Yes / No / Prefer not to answer

1. Since the age of 15, has anyone ever physically assaulted you? Physical assault can include throwing things at you that could hurt you, pushing or shoving you, hitting or kicking you or attacking you with an object.

Yes / No / Prefer not to answer

**Additional questions following a “Yes” response for each of questions 1 – 4 above.**

1. Has this happened to you more than once since the age of 15?

Yes / No

If yes, approximately how many times has this happened since the age of 15?

Twice / 3 to 5 times / More than 5 times

1. Thinking about this experience, or if it has happened more than once, the most recent incident:
2. Who was responsible for this incident and what was the relationship of this person to you?

- Partner or ex-partner
- Parent
- Son or daughter
- Brother or sister
- Other relative
- Stranger to me
- Friend or housemate
- Acquaintance or neighbour
- Employer/manager/supervisor
- Prefer not to say

1. What was the gender of the person responsible?

Male / Female / Other

1. Approximately how old were you?

- 15 to 17
- 18 to 24
- 25 to 34
- 35 to
- 45 or older

1. How did you view this incident at the time?

- As a crime
- Not a crime
- Something that just happens
- Unsure

1. Did you confide in anyone about what had happened to you at the time e.g., to friends, family members, work colleagues?

Yes / No / Can’t recall

1. Did you report this incident to police?

Yes / No / Can’t recall

1. What were your reasons for not contacting police? (Please tick all that apply)

- Felt ashamed or embarrassed
- Did not want the person responsible arrested
- Did not regard the incident as a serious offence
- Did not know or think the incident was a crime
- Did not think there was anything the police could do
- Did not trust the police
- Felt you would not be believed
- Fear of the person responsible
- Fear of legal processes
- Cultural reasons
- Communication difficulties

Other (please describe)

1. Did anyone else report this incident to police on your behalf? e.g., your parents or siblings.

Yes / No / Unsure

1. Was anyone ever charged with an offence in relation to this incident?

Yes / No / Unsure

1. Was anyone ever convicted of an offence in relation to this incident?

Yes / No / Unsure
